# Transactions of the Indiana State Medical Society at Its Eighth Annual Session, Held in the City of Indianapolis, May 19, 1857

**Published:** 1857-08

**Authors:** 


### BOOK NOTICES.

*Transactions of the Indiana State Medical Society, at its Eighth Annual Session, held in the City of Indianapolis, May* 19*th*, 1857.

This is a well printed pamphlet of 74 pages, containing an abstract of the proceedings of the Society at its late meeting; together with the reports of Committees, the Constitution and By-laws of the Society, and Names of Members.

The recent meeting seems to have been well attended, and highly interesting to the profession of that State.

From the reports we copy the following, which our readers will find well worth a perusal:

ON DISEASES OF THE EYE AND EAR, BY THEOPHILUS PARVIN, M. D., CHAIRMAN OF THE COMMITTEE.

Ophthalmic and Aural Pathology are connected by more than a conventional union. Analogies which obtain between light and sound, between the anatomy and physiology of the eye, and the anatomy and physiology of the ear, have counterparts in the morbid conditions to which the organs of vision and hearing are subject. Another bond of union is found in the fact that disease of the one organ may be associated with disease of the other, or may be metastatic with reference to it; nor are these facts at all surprising *a priori*, when we remember the continuity of structure belonging to the mucous membrane of the eye and lids, and that which lines the eustachian tube and internal ear; and moreover, that this membrane is continuous with that of the alimentary canal, and thereby a wide range of sympathetic action must be established, that may evince itself in disorder not only of either of these organs of special sense, but of both together, or of both alternately.

The essential character of an eye or of an ear is an expansion of nervous matter, excitement of which by any cause produces the sensation of *light* in the case of the former, of *sound* in that of the latter. For the perfection of each, so that one may be a complete optical instrument, the other a complete acoustic apparatus, certain subsidiary parts are essential; while nature, with consummate wisdom, provides for the protection of each by defences of bone and sentinel nerves.

We have much oftener to treat maladies located in one or more of those subsidiary parts than those belonging to the true eye or ear. In St. Mark's Hospital, during a period of eight years, among 2,385 patients there were but 114 cases of nervous deafness, while there were 553 of inflammation of the external meatus. In the Glasgow Eye Infirmary, during the year 1851, there were, of 1,048 patients, but 45 registered as having amaurosis, 156 with pure conjunctivitis, and in at least three hundred more the conjunctiva was affected with one or more of the other tissues of the ball, or of its appendages. In the Ophthalmic Hospital, at Canton, in the years 1850 and 1851, the entire number of patients being 3,759, diseases of the retina claimed but 165, while those of the conjunctiva had 1,356; and in the Royal London Ophthalmic Hospital, during a period of ten years, with 65,553 patients, the cases of amaurosis were but 4,865; and, on the other hand, conjunctival maladies numbered 35,193.

These statistics, taken at random from the records of various institutions, are introduced merely to present, in the most striking light, confirmation of the truth asserted—a truth which commands the immediate assent of every physician's knowledge and experience. This truth, however, does not diminish one tittle the demand for accurate diagnosis, and skillful and scientific treatment, when we reflect that disease of the secondary tissues may invade the nerve substance itself, or in its direct consequences be as damaging to the functions of the organ as if it had. Corneitis may terminate in retinitis, while the purulent ophthalmia have caused vastly more cases of blindness than have diseases of the retina. Deafness is much oftener consequent upon some malady of the meatus, or of the tympanal membrane, than of the auditory nerve.

In the investigation and in the treatment of diseases of the eye and of the ear, there are demanded not merely a thorough acquaintance with the anatomy of these organs, and a cultivation of the intellectual faculties, but likewise an education of touch, sight, and of hearing, too, since auscultation is resorted to as a means of diagnosis in aural pathology, and great dexterity, if not ambi-dexterity; nor should we omit from this catalogue of qualifications an accurate knowledge of optics and acoustics. Those who in this department have made themselves famous as practitioners and authors, have done it by laborious investigation, by patient observation, by studying, not merely books, but actual diseases, wherever found, and as often and under as varied circumstances as possible; indeed much must be learned in it as men learn a *trade*—by careful and continued practice.

It is unfortunately true, especially in our Western country, that very many of the people are neither just to themselves nor just to our profession, as it regards diseases of the eye and ear; and men in most respects intelligent, who, when assailed by some general disease, would shrink from the presence of a quack as from pollution and death, readily employ such a character when an ophthalmic or aural malady is to be relieved. If the people alone were to blame for the support given ignorant or designing quackery, we might leave them with sightless eyes, or with deaf ears, and empty pockets, to bemoan sorely their own folly, trusting that eventually there would be a reaction from this greatest evil, and they would learn that their truest friends and wisest counsellors in every physical malady were the members of the regular profession. But should we not ask ourselves, have we cultivated these specialties as we ought? Have we educated ourselves more particularly in the diagnosis of diseases of the eye and of the ear, and likewise in their treatment? Should not some change be made in the teaching of our medical colleges—as recently suggested in the Cincinnati Medical Observer by one of the ablest and most eminent members of our profession in the State (Dr. Byford, of Evansville)—so that ophthalmic and aural maladies, and other specialties, if deemed necessary, shall be more thoroughly and completely taught?

In the brief period allotted to student life in our institutions devoted to medical instruction, crowding professors and cramming students, and with the few facilities for personal inspection, and, alas! too, with the unsatisfactory and partial preliminary training of very many who aspire to the doctorate, or rather upon whom the degree descends, how few there must be who at the outset of their professional career are qualified to diagnose one-fourth of the diseases of the eye or ear! Yea, how many practitioners may be found whose ophthalmic pathology is embraced in the words "sore eyes," and whose entire treatment in diseases of the ear consists in syringing the external meatus with tepid water, or astringent solutions, or introducing into it sweet oil and laudanum, or various stimulants and irritants. Surely the facilities for acquiring both theoretical and practical knowledge in these most important branches of our profession should be greatly increased; the medical student should hear more and see more of the diseases of the eye and of the ear, and every physician should be as thoroughly qualified for their treatment as he now is not only for general practice, but likewise for the duties of surgeon or accoucheur. Were this done, the richest field of quackery would be wrested from the vile harpies who now occupy it so largely, and become the sole, undisputed and perpetual possession of legitimate medicine.

Having already spoken in somewhat general language of the diseases of the eye and of the ear, and also called attention to some of the more obvious relations which they sustain to our duties and interests, we propose more particular consideration of these diseases, merely in their therapeutical relations, endeavoring also to present a partial *resume* of recent important facts and truths pertinent to them.

Diseases of the ear have never commanded at the hands of the profession that study which is their due; for, in addition to the facts that the literature of the subject is but scanty, and the best of it of but recent origin, the study of the anatomical structure of the middle and external ear, still more of the changes induced by disease, is invested with many and serious difficulties. The function of hearing, too, is often impaired so gradually, and even its entire loss not being half such a calamity, either in reference to happiness or usefulness as the destruction of vision, the physician is not consulted until even the most intelligent treatment would often fail in effecting cure, or even relief. The testimony of one of the ablest writers upon diseases of the ear is, that aural surgery is now no farther advanced than ophthalmic surgery was fifty years ago.

The general principles of scientific medicine and surgery are undoubtedly our best guides in the study, examination and treatment of diseases of the ear. That important first step—diagnosis—is especially important in all aural diseases. Indeed it might be advisable, when the diagnosis cannot be satisfactorily made, to decline treating cases. For the exploration of the external ear, sunlight is undoubtedly preferable to any artificial light; and the polished tubular silver speculum will be found, as a general thing, much better than the valved instrument. Catheterism of the Eustachian tube is an operation which is not only dangerous, but is rarely necessary, and as rarely, perhaps, beneficial; therefore, the physician who attempts it should not only be satisfied of his skill, but likewise of the absolute necessity for the step. As a general thing, we use the same ointments and solutions in diseases of the external ear that we do in analogous diseases of the eye.

Among the recent novelties in aural practice may be mentioned a method of treating otorrhœa, proposed by Mr. James Yearsly, which consists in applying dry cotton, pressing it directly upon the suppurating surfaces, after having cleaned the meatus by gentle syringing and a porte sponge. Mr.

Yearsley's plan, however, receives decided condemnation from Mr. Toynbee, who, as you well know, is one of the very first authorities in aural diseases.

Mr. Westropp, of Bristol, proposes a new form of artificial membrana tympani: it is made by dipping an oiled stick, cut in the proper shape, into a chloroformic solution of gutta percha, repeating this until a film of sufficient thickness is formed to peel off in one uubroken piece. The membrane thus formed will be a tube closed at one end. This is found to answer a better purpose, in many cases, than the dry cotton plan of Yearsley, or the vulcanized india-rubber membrane of Toynbee.

The Ophthalmoscope marks a new era in the investigation of certain diseases of the eye. It should be a matter of pride to us that a native of our own State, Dr. E. Williams, now of Cincinnati, introduced this instrument in London—Dr. Dixon using his instrument in the Royal London Ophthalmic Hospital; and Dr. Williams' was among the first and most complete accounts of the ophthalmoscope, the method of using it, and the revelations made by it, published in the English language. The ophthalmoscope is now a *sine qua non* to every one who wishes to be completely prepared for the diagnosis of the various diseases of the eye. However beautiful in theory this exploration of the posterior segment of the ball, so that organic changes may be as accurately and fully known as if located in the cornea, may be, yet it must be confessed that, in practice, the ophthalmoscope is of more negative than positive value. Its use is prevented by several pathological conditions which may obtain in the anterior segment of the ball; and, on the other hand, the evil consequences which may result from its application forbid a rash resort to it. "The chief value of the ophthalmoscope," according to Mr. Dixon, "seems to consist in enabling the surgeon to set aside, as positively hopeless, a large number of cases formerly termed *amaurotic*, or nervous, which were assumed to be still curable, because their real nature could not be demonstrated."

The method of sub-conjunctival section in operating for strabismus, proposed by Guerin seven years since, is now meeting with considerable favor. Various forms have been given to the knife for cutting the tendon, while Mr. Critchett recommends blunt-pointed scissors. Just here we would remark that the boldest plagiarism we ever met with in medical literature has been recently perpetrated by either a man or a myth, under the name, titles, etc., of "Thomas Graham, M. D., F. R. C. S., etc., etc., late of Sidney, Australia." The original article is by Mr. Critchett, and is published in the London Lancet, May, 1856; while the plagiarism is published as an *original* article in the second number of the North American Medico-Chirurgical Review, March, 1857. It is strange that Professors Gross and Richardson could be so imposed upon.

The majority of ophthalmologists still adhere to the nitrate of silver treatment in the purulent ophthalmiæ, although there is great diversity in the strength of the solution resorted to, some using but one grain of the salt to the fluid ounce of water, while the greater number prefer from two to six, or eight, or even ten grains.

We have in the purulent affections of the conjunctiva a perversion of the normal function of that tissue. We know that the laryngeal and tracheal mucous membrane, in a certain form of inflammation, instead of elaborating its normal secretion, proceeds a step higher in the scale of vital actions, and exudes lymph. In like manner the degree of inflammation is such, combined with exposure to the atmosphere, that the conjunctiva produces a true puruleut secretion—exudation corpuscles are converted into pus-globules. Now in each of these morbid conditions the voice of intelligent experience declares unequivocally in favor of the efficacy of a solution of the nitrate of silver applied directly to the diseased surface; and possibly the philosophy of its action in each case is, that as an irritant or stimulant it induces a diseased condition inconsistent with that previously existing. It is not, however, all cases of conjunctivitis that should be treated with this remedy, but only those marked with decided purulent discharges. * * * *

In the treatment of the diseased condition of the conconjunctiva, generally of that portion belonging to the upper lid, commonly known as granular lids, or granular conjunctivitis, but which is really hypertrophy of the villous tissue, but little progress seems to have been made recently. As local applications to the morbid growth, escharotics are relied on by some, astringents by others, while most use both. The late Mr. Tyrrell, whose treatise on diseases of the eye cannot be too highly prized, was the first to introduce the mild plan of treating this disease, after having signally failed with the heroic measures pursued by Saunders; and certainly Mr. Tyrrell's success, mainly with the liquor plumbi sub-acetatis as a local application, is much in its favor.

Mr. Dixon speaks favorably of sugar of lead finely powdered and carefully dusted over the diseased surface, completely coating it and filling up all interstices. Dr. Jacob applies the same remedy to the inverted lid, but is careful to wash it off thoroughly. Mr. Dixon's mode of applying this remedy we believe to be palpably wrong. The treatment pursued by Jacob, along with other local remedies, such as sulphate of zinc and nitrate of silver in soluton, conjoined with constitutional treatment, may sometimes be very beneficial; yet it must be confessed that the acetate of lead, thus applied, often produces an alarming degree of inflammation, and we cannot counsel this treatment. One of our number has recently found, in several cases, as a first application to the granular surface, nitrate of copper of great advantage; it is less painful than the sulphate, and its results more satisfactory; it requires time and more numerous experiments to decide upon its value.

Among other new local remedies, the chloride of zinc, one grain to the fluid ounce, has the authority of Mr. Critchett; while Dr. Hays, in the last edition of Lawrence, recommends, in certain obstinate cases, a solution of iodide of zinc—a remedy which has recently received the commendation of two other distinguished members of the profession, without according credit to Dr. Hays for its original suggestion—*per contra*, the iodide of zinc has not proved satisfactory in Dr. Fenner's hands.

Dr. C. S. Fenner, of Memphis, Tennessee, highly eulogizes the *phytolacca decandra* as an internal remedy in the treatment of granular lids. He writes, in the first number of the North American Medico-Chirurgical Review: "With the aid of this remedy, I have been enabled to effectually cure cases of granular conjunctiva that, without it, would have resisted all my efforts; indeed, with me it has proved almost a specific for the exacerbations attending this complaint. Patients under the influence of phytolacca often expose themselves, and take a severe cold, without affecting the eyes in the least." Dr. Parry, of Indianapolis, some time before the publication of Dr. Fenner's paper, made use of the poke root in the case of a patient who had an attack of rheumatism while laboring under a severe and obstinate conjunctivitis; in a short time the rheumatism yielded, and likewise the conjunctival inflammation, though this had previously resisted ordinary treatment.

Without constitutional treatment of some kind, we doubt whether any local means would alone effect a cure where there is a decided tendency to the production of large, spongy, bleeding granulations; nor do we believe that any of the various remedies resorted to approximate the character of a specific. Among constitutional remedies we believe that tonics, especially quinine and iron, occupy the first rank.

A European physician has recently found great benefit in two cases of marked albugo, from the use of galvanism; he also found this remedy very useful in four cases of nervous palpebral palpitation. "In the first, persistent convulsive movements of the eye-lid, accompanied by great photophobia, had resisted other means. In the second, involuntary contraction, due to bad acquired habits, occurring in a girl ten years of age, were relieved. The third was another example of convulsive motion of the eye-lid, with photophobia; and the fourth was a case of ptosis."

Dr. Henry W. Williams, in a paper read before the Boston Society for Medical Observation, last August, and subsequently published in the Boston Medical and Surgical Journal, advocates the non-mercurial treatment of iritis, and adduces forty-eight cases, in the treatment of which no mercury was used, the result being uniformly favorable, except in four who had been injured by irregular practice. "The remedies mainly relied upon"—we quote from the Cincinnati Medical Observer—"were atropia, ten grains to the ounce, as a local application; iodide of potassium, quinine, opiates at night, and occasional leeches." The quantity of atropia is extravagantly and unnecessarily large; indeed, a solution of that strength would be highly irritating; and moreover, its application might be followed by fatal consequences. No doubt the iodide of potassium is the great curative agent in the treatment pursued by Dr. Williams—this remedy and turpentine ranking next in efficacy to mercury in iritis. Notwithstanding the favorable results following Dr. Williams' plan in the cases he reports, yet the contra-indications to the use of mercury must be very decided, before we would risk a case of acute inflammation of so important a structure as the iris, to such treatment.

A few years since Mr. Canton stated, in the London Lancet, that the *arcus senilis*, often seen in cataractous patients, though not exclusively in such, was a fatty degeneration, "and in no instance was this condition observed without there being fatty degeneration of the heart." Here he seems to have rested his investigation, not examining whether the opacity which may invade the lens in the aged might not, sometimes at least, be similar in cause and character to that affection of the cornea, the connection of which with cardiac disease he so clearly demonstrated. In the April number of the British and Foreign Medico-Chirurgical Review, Mr. Jordan, Demonstrator of Anatomy at the Queen's College, Birmingham, adduces nineteen cases of cataract, every one having disease, more or less serious, of the heart; "and in several of the cases a fatty condition of the heart might be reasonably predicted." Microscopic examination of an extracted cataract revealed fat globules in the nuclei of the delicate cells covering the surfaee of the crystalline lens, and here and there a few delicate plates of cholesterine." If the question, "May not cataract be the result of a process identical with or analogous to fatty degeneration?" asked by Mr. Jordan, be answered in the affirmative, much light will be thrown on one of the obscure points in ophthalmic pathology, for certainly the nature and causes of simple, uncomplicated cataract have hitherto been but very partially known. As to the relation between heart disease and cataract, the writer would state that a few weeks since he operated for cataract upon a patient who presented decided evidence of disease of the heart. The patient—a female—was 63 years old, and had been remarkably healthy until within a few months, when she suffered from severe headaches and attacks of fainting, if she exerted herself much, or suddenly assumed an erect position, and her vision commenced failing in the right eye—the sight in the left was lost twenty years before from neglected iritis—a distinct *arcus senilis* marked each eye.

ON VERATRUM VIRIDE, BY P. H. JAMESON, M. D., MEMBER OF THE COMMITTEE.

Few articles of the Materia Medica are at present more the subject of thought and discussion among medical men, particularly in the South and West, than *Veratrum Viride.* This agent was known as a powerful remedy by the aborigines. Prof. Tully, of Yale College, is said to have used it for many years in his practice, and recommended its use in his lectures to his medical classes.

His student, Dr. Osgood, of Providence, in an essay published in the August number of the American Journal of Medical Science, in 1835, gives an account of many of its effects on the human system, particularly its sedative influence on the heart's action, and its want of cathartic power, unlike its congener, Veratrum Alba.

About six or seven years ago, the attention of medical men was again called to this remedy by Dr. W. C. Norwood, of Cokesbury, South Carolina; since which time few remedies have so speedily attained such prominence—not so much in books and journals as in the unwritten materia medica of the practical physician.

I have used this agent frequently within the last three years, and regard it as one of great power and certainty, and applicable to many of the forms of disease which are constantly occurring. With Dr. Wood, I consider Veratrum Viride a nervous sedative, which acts directly on that part of the nervous system governing the circulatory function, and not indirectly through a nauseant impression, as supposed by some observers. There is often a marked diminution of the frequency of the pulse, without the least nausea, as I have frequently noticed; and, under similar circumstances, Dr. Norwood has observed its reduction to thirty-five beats in a minute. On the contrary, there is sometimes nausea without the circulation being affected.

These circumstances favor the idea that the emetic qualities of Veratrum Viride are incidental, possibly dependent upon the existence of several primary elements, to which its nauseant and sedative properties are respectively referable. Dr. Norwood claims that this remedy is alterative, or deobstruent, in a degree; also, an expectorant, diaphoretic, nervine, emetic, and an arterial sedative; at the same time disclaiming for it any narcotic properties whatever. It is not improbable that most of the effects here claimed are the results of its sedative action. Arterial excitement is not favorable to secretion, or the due performance of the function of any organ; and its reduction is likely to be followed by a more healthy action. I have administered this remedy in pneumonia, typhoid and puerperal fevers, with very satisfactory results.

In pneumonia it is an agent of great power and utility. It is needless to discuss the pathology of this disease minutely. When there is a disproportion between the breathing surfaces and the quantity of blood thrown into the vessels of the lungs, respiration, a great vital function, is at once impaired, and each extra stroke of the heart increases the difficulty. To relieve this condition, when the constitution will admit of it, the intelligent practitioner makes haste to employ the lancet, antimony or mercury, singly or in combination, for the purpose of quieting the heart and changing the quality of the circulatory fluid, by lessening its inflammatory properties. After a free blood-letting, who has not seen his pneumonic patient breathe with that intense satisfaction with which the thirsty man quaffs the pure water, or express himself as comfortable when brought under the intolerable nausea of tartar emetic.

Veratrum Viride is a remedy which I have often seen produce these salutary effects in a marked degree, without loss of blood, nausea, or salivation, which quiets the heart, increases expectoration, and indirectly restores the depurating functions of the liver and kidneys. In different cases it may be given with benefit either after blood-letting or along with quinine. A good mode of prescribing it for the adult is about this:

℞ Tinct. Verat. Vir. (Norwood's), f. ʒi.

Nit. Potass. Pur., ʒi.

Aqua, ℥i.

M. A teaspoonful every four hours, to be alternated with small doses of mercury, if deemed advisable. This treatment alone, continued for twelve or eighteen hours, I have seen produce most satisfactory results, the patient's condition being very similar to that observed, in such cases, after a copious blood-letting. The pulse will be softened, and have come down from a hundred, or a hundred and ten, to seventy or eighty; and this, in many cases, without nausea. If the remedy fails, the dose may be increased gradually till it is nearly doubled: this is seldom necessary. It is wrong, and hazardous, I think, to force the pulse down at once by full doses; and needless to reduce it below what, in a given case, may be considered a normal standard as to frequency. A greater reduction than this must in some degree interfere with the due performance of the functions of the system generally; and to cause it is to tamper with a remedy whose power may not be fully understood. After the first twenty-four or forty-eight hours, the dose may be diminished one-third, one-half, or even more, unless the pulse should rise, and the symptoms grow worse; such being the case, the dose should be increased.

One considerable advantage which this remedy possesses over tartar emetic is, that it does not purge, which is often a serious objection to the use of antimonials in pneumonia. I have administered veratrum viride, in many cases of pneumonia, to patients of all ages and both sexes, and I am confident that the average duration of the attack has been shorter, and the patient has suffered less from the disease, than under any other mode of treatment which I have witnessed.

Verat. vir. is also a good remedy in many cases of typhoid fever, especially where there is a dry, hot skin, parched, dark-brown tongue, and a quick, sharp pulse, with sleeplessness. Some of you will doubtless recollect that Dr. Graves recommends tartar emetic, in such cases, to procure rest. Six drops may be given every four hours, combined with nit. pot., and if there is irritability of the stomach or bowels, a little morphine may be added. If the pulse is not reduced in twenty-four hours, the dose may be increased. When the pulse is as low as seventy, in the adult, I do not attempt a further reduction. Under this treatment the skin becomes moist and cool, the urinary secretion increased, and the tongue is less dry; indeed, all the symptoms of the disease are mitigated, and in some cases, I have no dobut, the duration of the fever is shortened. It is but fair to add, that in both typhoid fever and pneumonia I have sometimes, though rarely, administered verat. vir. so as to produce nausea, without the least impression upon the circulation, and, consequently, with no benefit to the patient. This remedy is valuable, not only in such cases of typhoid fever as before referred to, but in all cases where the pulse rises above one hundred, whether attended with restlessness or stupor.

I have employed verat. vir. in two cases of puerperal fever, with what I consider favorable results.

The first of these cases occurred at a time when puerperal fever was quite prevalent and fatal. The effect of the verat. vir. during the first four days of treatment was to reduce the pulse from 140 to 76 beats per minute, almost without nausea or vomiting. What seemed remarkable was, that the pain was greatest when the pulse was least frequent. This may have been owing to the return of vitality, under the influence of the remedy, for it is well known that want of vitality in the affected structures sometimes renders this disease almost painless. Calomel, opium, and other remedies were freely employed; still, I believe that if the patient owed her recovery to the treatment, the credit was mainly due verat. vir.

A very rapid pulse is characteristic of puerperal fever; it is generally small, because the great current of the blood is diverted from its natural channel, and thrown on to the weakened and congested capillaries of the inflamed peritoneum. These vessels cannot unload themselves until the heart's action is reduced. To accomplish this end, Dr. Gordon, of the last century, Dr. Robert Lee, the younger Baudolocque, Dr. Meigs, and many others, advise us to bleed early and freely, and repeat as often as the pulse rises. However scientific this treatment may be, I believe it does not meet with the gêneral approval of Western physicians. Bleeding modifies the character of the blood by lessening its vitality, which, in this locality, is not always desirable.

With the verat. vir. the pulse can be as much reduced in frequency, and kept down as long as the practitioner desires. With it, puerperal fever may be, if not arrested at its outset, modified in its course, and effusion, that most fatal of its consequences, in many cases prevented.

The question arises in the mind of the practitioner, whether verat. vir., acting with such power and certainty on the heart, is a safe remedy. Digitalis, very like it in some respects, is known to be poisonous, and few administer it without a certain degree of caution. In Dr. Wood's new work is given a statement of Dr. Osgood, that the farmers of New England, to protect their crops from birds, were in the habit of scattering in their fields corn which had been soaked in an infusion of

American hellebore. The birds, after eating this grain, became paralyzed, and in this condition were readily caught; but, if not disturbed, the effect soon passed off, and they flew away unharmed.

Dr. Wood also states that he has seen no account of fatal poisoning from the use of this agent. Prof. Winston, of the Medical Department of the University of Nashville, in a paper read before the State Medical Society of Tennessee, says: "The most remarkable, and at the same time the most important effect of verat. vir. is, the reduction of the heart's action, and that, too, with entire safety to the patient." His position and locality are certainly favorable for observing on this subject. But the foregoing testimony, however high, is negative. Dr. W. A. Brown, in a paper published in the October number of the Nashville Medical Journal for 1856, contends with great vehemence that this agent is an abortive, and speaks incidentally of its poisonous qualities. His paper, claiming to be a review of Prof. Winston, contains no case or fact shedding much light upon the subject, and can only be regarded as the opinions of Dr. Brown, expressed in a very decided manner. The idea is not unreasonable, that a medicine which makes a profound impression on the nerves of organic life, may so far interfere with the functions of the uterus as to cause abortion, although I have witnessed no such effect from veratrum viride.

During the last winter, at a meeting of the Indianapolis Medical Association, verat. vir. was incidentally discussed. One of our most prominent practitioners, whose opinions deserve high consideration, stated that he had seen a little patient sink under the depressing influence of that agent; the pulse was reduced to 35 or 40, and the child died. Considering the case as almost hopeless, he had given the medicine as a last resort.

A patient in my care, affected with pneumonia, died rather singularly. In conclusion, I shall venture to report this case from memory, and leave you to make your own inferences. The patient was a rather delicate lady, about 30 years of age; but, as I learned from her husband, subject to hysteria. She was severely attacked with pleuro-pneumonia, was very restless, suffered acutely, and was uncommonly nervous. I gave her veratrum vir. in small doses once in four hours, along with other remedies. When I visited her on the morning of the fifth day, she was sitting up; she greeted me with a laugh, said she had passed a pleasant night, and seemed to think herself about well. I discovered nothing remarkable in her condition, except a little nervousness and coldness of the extremities. She was directed to return to bed, a cordial prescribed for her, and I left her without much concern. I was hastily called again about noon, when I found her in a most alarming condition; the head was thrown back, the eyes turned up, the power of deglutition had failed, and breathing was performed with the utmost difficulty. There was a bloody froth about the mouth. The pulse, weak and irregular, was about 120. The extremities were cold, but there was great heat about the head and chest. As to treatment, all was done that could be, in the way of external remedies, and enemata; but the patient died that night at 10 o'clock. There was no *post mortem* examination, but I supposed the immediate cause of death to have been congestion of the lungs, and about the heart. She had been affected with ague the previous autumn.

Unless corroborated by other cases, it would be hasty and illogical to conclude that this result was the sole effect of veratrum viride. I lately saw an account of the death of a surgical patient from congestion of the lungs, in a locality, where, I presume, this agent was never heard of. The previous malarious impression, or imprudent sitting up, were the more probable causes of this fatal congestion. The veratrum might possibly have acted in conjunction with these causes; and if the mere suspicion will serve to put others on their guard, as it did me, I shall have attained my object in referring to this case.

Sometimes, though very rarely, verat. vir. produces nausea and vomiting, with great depression; but I have seen worse effects, and harder to correct, from antimony. There are sometimes sensations of choking or smothering, and difficulty in breathing; or there may be hiccough or dizziness—all of which soon pass away when the use of the remedy is suspended. Like mercury, antimony, opium, quinine or chloroform, verat. vir. is not only powerful for good, but doubtless, in unskillful hands, sometimes potent for evil. That which can do no harm seldom does any good. A misstroke of the surgeon's knife may destroy the life of his subject. As that surgeon approximates most nearly to perfection in his calling who knows not only how, but when, to use the knife, so that physician attains the highest degree of excellence in his profession who can secure to his patient the good without the evil effects of a powerful agent, and know when it is required. In this consists the distinction between the true physician and the charlatan.

The report on Medical Education will be noticed in another place.

